# Single-cell and intercellular communication analyses reveal DLL3 as a key regulator in the glioma tumor microenvironment

**DOI:** 10.1016/j.omtn.2026.103099

**Published:** 2026-09-15

**Authors:** Lei Xie, Han Wang, Xianrun Pan, Zhaoxi Yang, Jing Hu, Yuncong Zhang, Hao Qian

**Affiliations:** 1 School of Medicine, University of Electronic Science and Technology of China, Chengdu 611731, China; 2 School of Life Science and Technology, University of Electronic Science and Technology of China, Chengdu 611731, China; 3 Guangdong Provincial Key Laboratory of Tumor Interventional Diagnosis and Treatment, Zhuhai Institute of Translational Medicine, Zhuhai People’s Hospital (The Affiliated Hospital of Beijing Institute of Technology, Zhuhai Clinical Medical College of Jinan University), Zhuhai 519000, China

**Keywords:** MT: bioinformatics, glioma, tumor microenvironment, intercellular communication, DLL3, cell cycle, cell proliferation

## Abstract

Glioma progression is increasingly recognized as a process driven not only by intrinsic genetic alterations in tumor cells but also by dynamic interactions within the tumor microenvironment (TME). However, systematic insight into the remodeling of intercellular communication networks during glioma evolution and their role in tumor progression remains limited. We integrated large-scale single-cell RNA sequencing with computational inference of ligand-receptor interactions to characterize the cellular landscape and communication architecture across disease stages. Single-cell analysis of normal brain and glioma tissues revealed pronounced cellular heterogeneity and stage-dependent remodeling of the TME, involving malignant cells, immune populations, glial cells, and vascular components. Intercellular communication analysis showed extensive reconstructing and strengthening of signaling networks during glioma progression, particularly among glioma cells, myeloid cells, astrocytes, and endothelial cells. Integrative analysis identified six communication-associated genes (*DLL3*, *ADCYAP1R1*, *LFNG*, *FGFR3*, *VEGFA*, and *EGFR*) that were significantly associated with patient prognosis. Functional validation further demonstrated that *DLL3* is essential for glioma cell viability and proliferation. *DLL3* depletion induced broad transcriptional reprogramming and suppressed cell cycle-related pathways, suggesting that *DLL3* may mediate the link between microenvironmental signaling and intrinsic proliferative programs. Together, these findings highlight intercellular communication as a central driver of glioma progression and a potential therapeutic target.

## Graphical abstract

**Figure:**
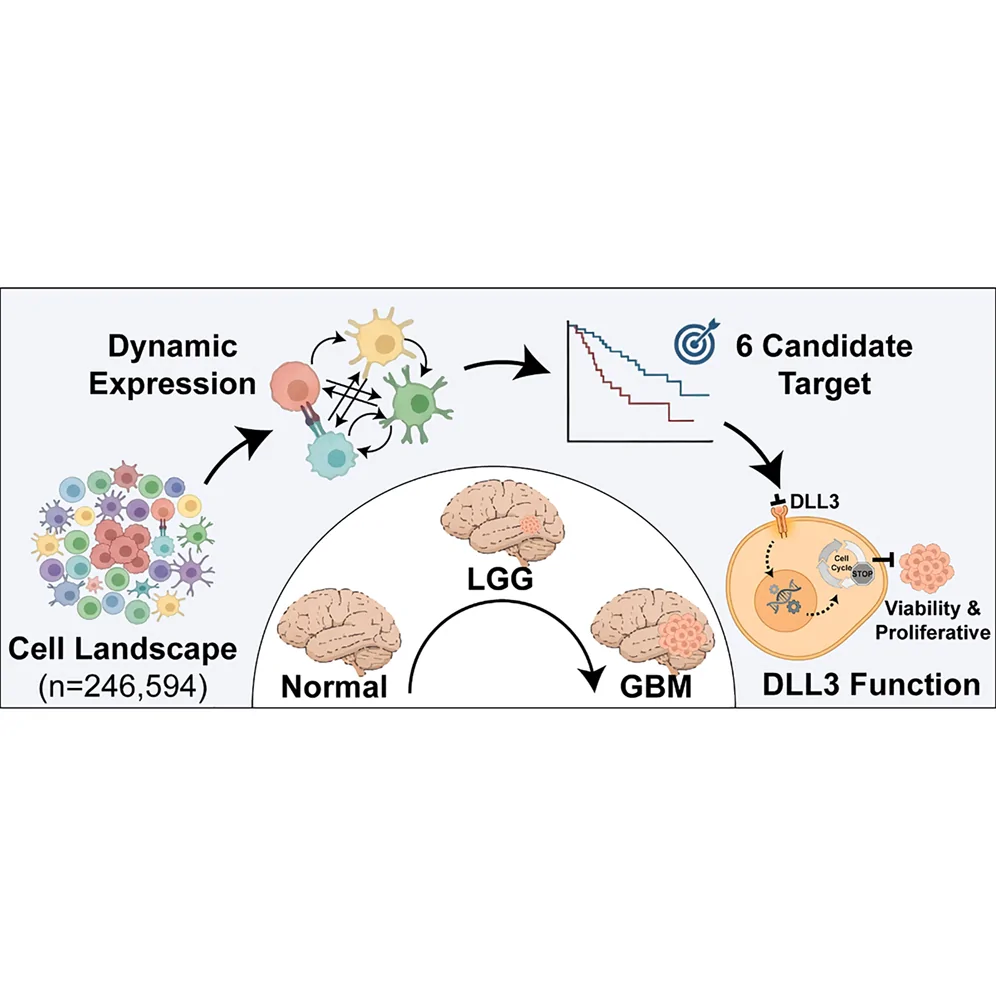

## Teaser

Glioma progression is driven by dynamic remodeling of tumor microenvironmental communication networks. Through integrative single-cell transcriptomic analysis, the authors systematically characterize stage-specific intercellular signaling alterations and identify DLL3 as a communication-associated regulator linked to proliferative reprogramming, providing insight into microenvironment-driven mechanisms and potential therapeutic targets in glioma.

## Introduction

Gliomas are the most common primary malignant tumors of the central nervous system and remain among the most lethal human cancers despite advances in surgical resection, radiotherapy, and chemotherapy.^1^^,^^2^^,^^3^ In particular, glioblastoma (GBM) is characterized by rapid growth, diffuse infiltration, and profound resistance to conventional therapies, resulting in dismal patient outcomes.^4^^,^^5^ Increasing evidence indicates that these aggressive features cannot be fully explained by intrinsic genetic alterations in tumor cells alone. Instead, the tumor microenvironment (TME), which consists of immune cells, vascular cells, glial cells, stromal components, and extracellular matrix, plays a decisive role in tumor progression, therapeutic resistance, and disease recurrence.^6^^,^^7^ In glioma, the TME is not a passive bystander but an active participant that dynamically interacts with tumor cells to promote proliferation, invasion, immune evasion, and metabolic adaptation.^8^^,^^9^

Recent studies have revealed that intercellular communication within the glioma microenvironment is a critical driver of tumor growth and progression.^10^ Beyond classical immune-tumor interactions, emerging evidence highlights the importance of neuron-glioma and glial-glioma cross-talk in regulating malignant behavior.^11^^,^^12^^,^^13^ Notably, neuronal activity-dependent secretion of neuroligin-3 (*NLGN3*) has been shown to stimulate glioma proliferation through activation of PI3K-Akt-mTOR signaling pathway, thereby establishing a direct functional link between neuronal circuits and tumor growth.^14^^,^^15^^,^^16^ In parallel, complex bidirectional interactions between glioma cells and immune populations, including microglia, macrophages, and T cells, contribute to the establishment of an immunosuppressive niche that promotes tumor survival and limits therapeutic efficacy.^17^^,^^18^^,^^19^ These findings underscore that glioma progression is orchestrated by a highly interconnected cellular network, in which diverse cell types exchange signals through ligand-receptor interactions, cytokines, growth factors, and extracellular vesicles.

Despite these insights, most existing studies of glioma biology have relied on bulk transcriptomic profiling, which captures averaged gene expression signals across heterogeneous cell populations.^20^^,^^21^ While bulk RNA sequencing (RNA-seq) has provided valuable insights into molecular subtypes and prognostic signatures, it inherently masks cellular heterogeneity and obscures cell type-specific signaling programs. This limitation is particularly critical in glioma, where malignant cells coexist with a diverse array of immune and non-immune cell populations, populations exhibiting distinct functional states and dynamic interactions.^22^ Consequently, bulk approaches are insufficient to resolve the context-dependent signaling events and intercellular communication processes that drive glioma progression.

Single-cell RNA-seq (scRNA-seq) has emerged as a powerful approach for overcoming these limitations by enabling high-resolution dissection of complex cellular ecosystems.^23^^,^^24^ By profiling gene expression at the single-cell level, scRNA-seq allows the identification of rare cell populations, characterization of cellular states, and reconstruction of developmental and functional trajectories.^25^^,^^26^ Importantly, integration of scRNA-seq data with computational frameworks for cell-cell communication analysis now enables systematic inference of ligand-receptor interactions and signaling networks among distinct cell types within the TME.^27^^,^^28^^,^^29^ This integrative strategy provides an unprecedented opportunity to map the communication architecture of glioma and to uncover key regulatory nodes that drive malignant behavior.^30^ However, despite the growing application of scRNA-seq in glioma research, a comprehensive and stage-specific characterization of intercellular communication networks in the glioma microenvironment remains lacking. Most studies have focused on specific cell types or individual signaling pathways, without systematically comparing communication patterns across different disease stages or integrating communication signals with transcriptional reprogramming and clinical relevance.^31^^,^^32^^,^^33^ Consequently, the global organization of cellular cross-talk in glioma and its contribution to tumor progression are still poorly understood.

In this study, we sought to systematically dissect the cellular landscape and intercellular communication networks of the glioma microenvironment through integrated scRNA-seq analysis with computational inference of cell-cell signaling. By analyzing single-cell datasets from multiple glioma cohorts and disease stages, we characterized dynamic alterations in cellular composition and communication patterns during glioma progression. Through integrative analyses of ligand-receptor interactions, differential gene expression, and patient survival data, we identified six communication-associated genes associated with glioma malignancy. Furthermore, we performed functional validation of *DLL3*, a key candidate identified through our analysis, and demonstrated its critical role in regulating glioma cell proliferation and transcriptional programs. Together, our findings provide a systematic view of intercellular communication in the glioma microenvironment and reveal *DLL3* as a functional regulator of glioma progression.

## Results

### Single-cell landscape reveals extensive cellular heterogeneity in the glioma TME

To comprehensively characterize the cellular composition of the glioma TME, we integrated four scRNA-seq datasets comprising normal brain tissues, LGG, and GBM samples. After stringent quality control and batch effect correction, a total of 246,594 high-quality cells was retained for downstream analyses (Figures 1A and 1B). Unsupervised clustering followed by annotation based on canonical marker genes identified eight major cell populations, including myeloid cells, glioma cells, T cells, oligodendrocytes, neurons, vascular cells, endothelial cells, and B cells (Figures 1C and 1D).

**Figure 1:**
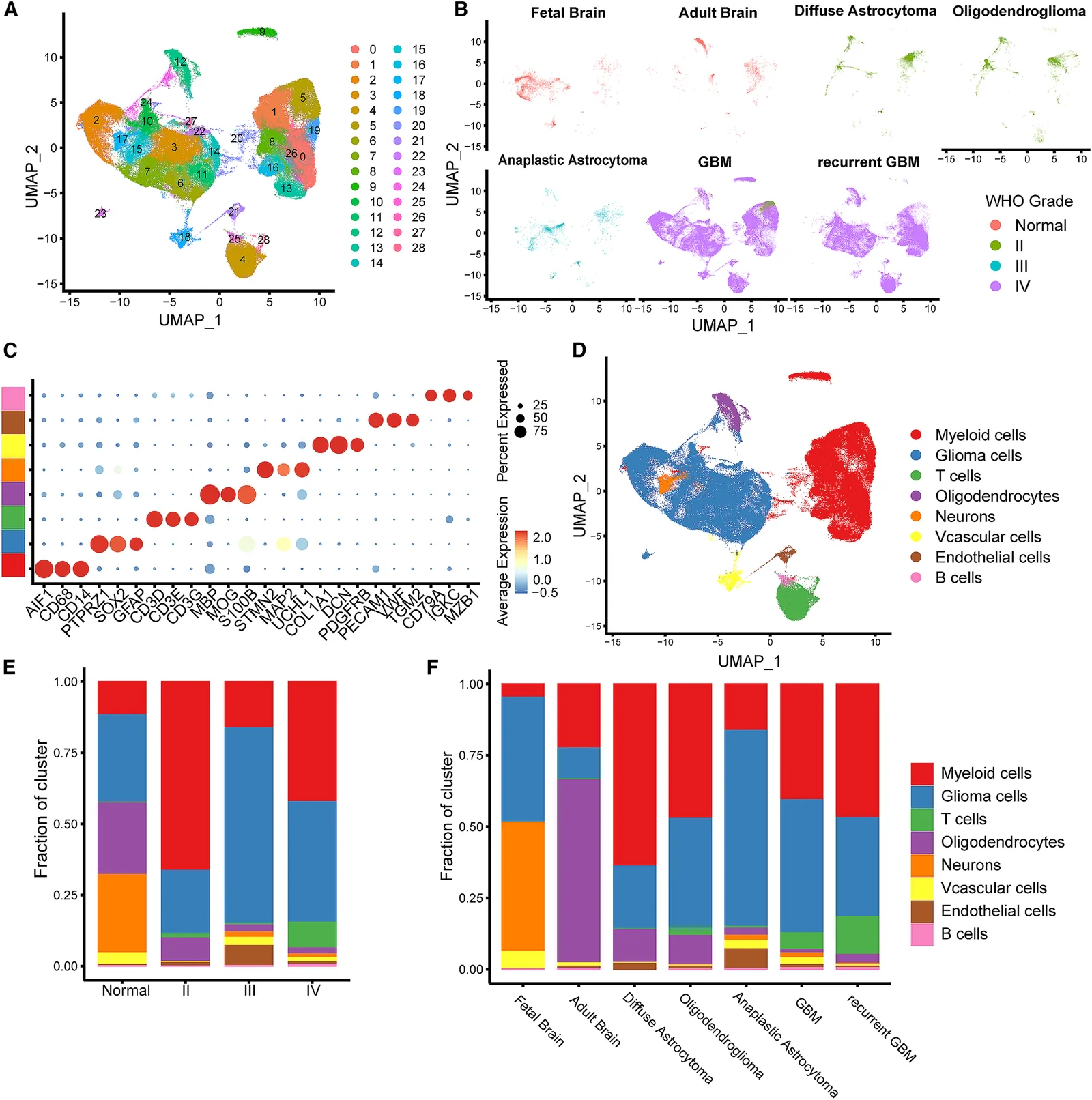
Single-cell transcriptomic profiling delineates cellular composition and dynamic changes across normal brain and glioma grades (A) UMAP visualization of integrated single-cell transcriptomes showing 28 distinct clusters across all samples. (B) UMAP plots of cells stratified by tissue origin and WHO grade, including fetal brain, adult brain, diffuse astrocytoma, oligodendroglioma, anaplastic astrocytoma, primary GBM, and recurrent GBM. (C) Dot plot showing canonical marker gene expression used for annotation of major cell types, including myeloid cells (*AIF1*, *CD68*, and *CD14*), glioma cells (*PTPRZ1*, *SOX2*, and *GFAP*), T cells (*CD3D*, *CD3E*, and *CD3G*), oligodendrocytes (*MBP*, *MOG*, and *S100B*), neurons (*STMN2*, *MAP2*, and *UCHL1*), vascular cells (*COL1A1*, *DCN*, and *PDGFRB*), endothelial cells (*PECAM1*, *VWF*, and *TGM2*), and B cells (*CD79A*, *IGKC*, and *MZB1*). (D) UMAP representation colored by 8 major cell types. (E) Stacked bar plots showing the fraction of each cell type across WHO grades (Normal, stage II, stage III, and stage IV). (F) Stacked bar plots showing the fraction of each cell type across different pathological entities, including fetal brain, adult brain, diffuse astrocytoma, oligodendroglioma, anaplastic astrocytoma, GBM, and recurrent GBM.

We next examined the distribution of these cell types across normal tissues and different WHO grades of glioma. Compared with normal brain tissues, glioma samples exhibited marked alterations in cellular composition, particularly in myeloid cells, glioma cells, and endothelial cells (Figure 1E). Notably, the proportions of these cell populations varied substantially across different tumor stages (II, III, and IV), indicating dynamic remodeling of the TME during glioma progression. Consistent trends were also observed when comparing different disease entities, including diffuse astrocytoma, oligodendroglioma, anaplastic astrocytoma, GBM, and recurrent GBM (Figure 1F). These findings highlight profound cellular heterogeneity and stage-dependent reorganization of the glioma microenvironment.

### Re-clustering uncovers diverse malignant and glial cell states during glioma progression

To further resolve the heterogeneity of *CD45*-low/negative cellular populations within the glioma microenvironment, we extracted 119,232 cells with low or absent *CD45* expression and performed re-clustering analysis. This analysis identified 12 distinct cell populations, including microglia, endothelial cells, vascular cells, ependymal cells, neurons, oligodendrocyte progenitor cells, oligodendrocytes, reactive astrocytes, inhibitory astrocytes, dividing glioma stem cells/neural stem cells (GSCs/NSCs), and GSCs/NSCs (Figures 2A and 2B). Cell type annotation was supported by the expression of established canonical markers, including *NES*, *SOX2*, *AQP4*, *SLC1A3*, and *EGFR* for GSCs/NSCs (Figure 2C). We then analyzed the distribution of these cell populations across different WHO grades and pathological conditions. Significant shifts in the proportions of oligodendrocytes, neurons, endothelial cells, and oligodendrocyte progenitor cells were observed across glioma progression (Figure 2D). Similar trends were evident across fetal brain, adult brain, diffuse astrocytoma, oligodendroglioma, anaplastic astrocytoma, GBM, and recurrent GBM (Figure 2E), highlighting dynamic changes in cellular states during glioma evolution. To distinguish malignant from non-malignant cells, copy number variation (CNV) inference was performed using CopyKAT. Aneuploid cells were classified as malignant, whereas diploid cells were considered non-malignant (Figure 2F). Quantification of malignant and non-malignant cells across tumor stages revealed substantial differences in their relative proportions (Figure 2G), further supporting progressive malignant expansion and microenvironmental remodeling during glioma progression. Collectively, these results demonstrate that both immune and non-immune compartments of the glioma TME undergo extensive reorganization across disease stages, suggesting that intercellular communication networks are likely to be dynamically reprogrammed during tumor progression.

**Figure 2:**
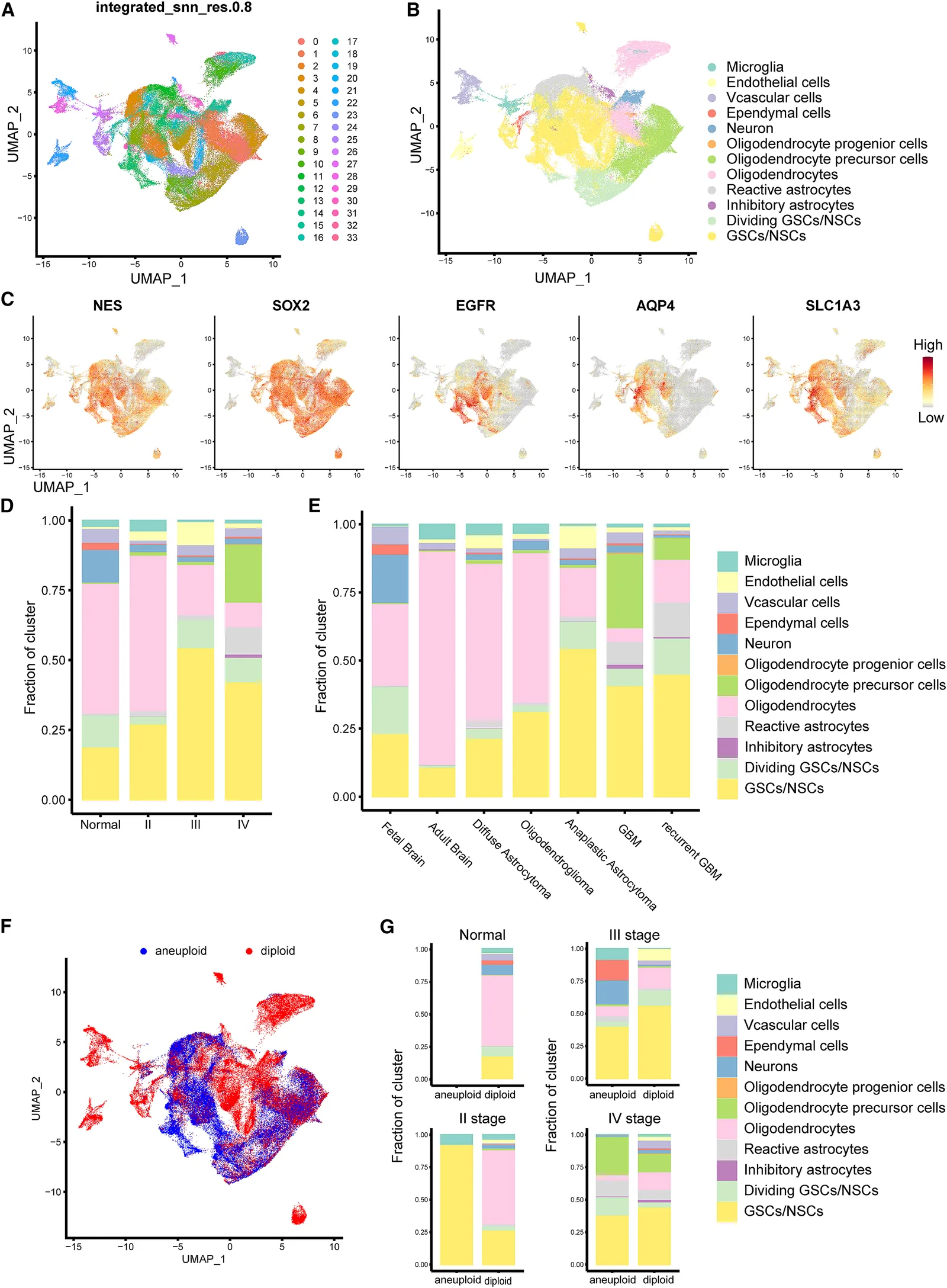
Cellular heterogeneity and stem-like states in glioma revealed by single-cell analysis (A) UMAP visualization of integrated glioma cells clustered at resolution 0.8. (B) UMAP plot showing annotated cell types, including microglia, endothelial cells, vascular cells, ependymal cells, neurons, oligodendrocyte progenitor cells, oligodendrocytes, reactive astrocytes, inhibitory astrocytes, dividing GSCs/NSCs, and GSCs/NSCs. (C) Feature plots showing the expression of GSCs/NSCs associated markers, including *NES*, *SOX2*, *EGFR*, *AQP4*, and *SLC1A3*. (D) Stacked bar plots showing the distribution of different cell types across WHO grades. (E) Stacked bar plots showing the distribution of different cell types across fetal brain, adult brain, diffuse astrocytoma, oligodendroglioma, anaplastic astrocytoma, GBM, and recurrent GBM. (F) UMAP visualization colored by inferred copy number variation (CNV) status, distinguishing aneuploid malignant cells from diploid non-malignant cells. (G) Stacked bar plots showing the proportion of aneuploid and diploid cells across WHO grades.

### Intercellular communication networks are extensively rewired during glioma progression

Given the pronounced changes in cellular composition, we next investigated whether intercellular communication networks were also remodeled during glioma progression. Using CellCall,^34^ we inferred ligand-receptor-transcription factor (L-R-TF) interactions among different cell populations in each tumor stage. As shown in Figures 3A–3D, both the number and strength of intercellular interactions exhibited marked differences across normal tissues and glioma stages II, III, and IV (Table S1). In normal brain tissues, intercellular communication was relatively sparse and organized, whereas glioma samples displayed increasingly complex and intensified communication networks with tumor progression. Notably, interactions involving glioma cells, myeloid cells, astrocyte populations, and vascular cells were markedly enhanced in advanced stages, indicating heightened cross-talk among malignant and stromal compartments. These findings demonstrate that glioma progression is accompanied not only by changes in cellular composition but also by extensive rewiring of intercellular signaling networks, supporting the notion that dynamic cell-cell communication is a key driver of TME remodeling and tumor evolution.

**Figure 3:**
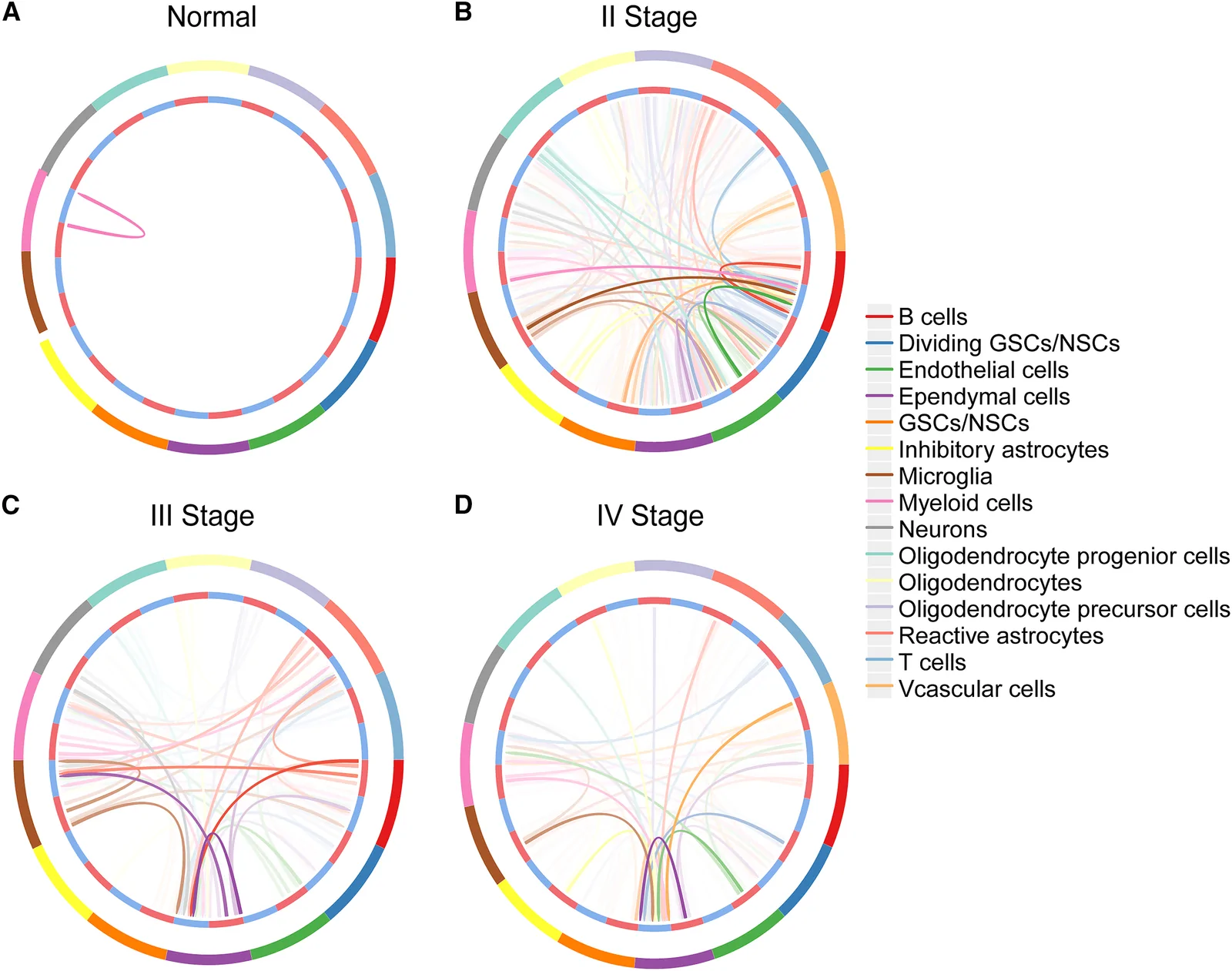
Progressive rewiring of intercellular communication networks across glioma grades (A–D) Circos plots depicting global ligand-receptor interaction networks in normal (A), WHO grade II (B), WHO grade III (C), and WHO grade IV (D) samples. Each colored segment represents a cell type, and connecting lines indicate predicted intercellular signaling interactions. The thickness and density of connections reflect the overall communication strength between cell populations.

### Integration of differential expression and communication analyses identifies six key genes associated with glioma progression

To identify molecular regulators associated with stage-specific communication patterns, we performed differential expression analysis across different tumor stages using DESeq2. The 407 stage-specific differentially expressed genes (DEGs) of GSCs/NSCs were identified, with 156 DEGs at the normal stage, 83 at stage II, 105 at stage III, and 63 at stage IV of glioma (Figure 4A; Table S2). Functional enrichment analysis using Metascape revealed that these DEGs were significantly enriched in biological processes related to RNA splicing, spindle organization, gliogenesis, nervous system development, synaptic signaling, and vascular processes (Figure 4B), reflecting active remodeling of both neural and stromal programs during glioma progression. We then intersected all stage-specific DEGs with genes involved in ligand-receptor interactions derived from the CellCall analysis. This integrative approach yielded six overlapping genes: *DLL3*, ADCYAP1R1, *LFNG*, *FGFR3*, *VEGFA*, and *EGFR*. These genes were further examined using GEPIA based on TCGA and GTEx datasets. All six genes exhibited significant differential expression between tumor and normal tissues in both GBM and LGG (Figure 4C). Kaplan-Meier survival analysis demonstrated that the expression levels of these genes were significantly associated with patient prognosis (Figure 4D). High expression of *DLL3* and *ADCYAP1R1* was associated with improved survival, whereas elevated *LFNG*, *VEGFA*, and *EGFR* expression correlated with poorer outcomes. *FGFR3* also displayed prognostic relevance, albeit with distinct patterns. Taken together, these results indicate that the six identified genes are closely linked to intercellular communication, transcriptional dysregulation, and clinical outcomes, suggesting their potential roles as key regulators of glioma progression.

**Figure 4:**
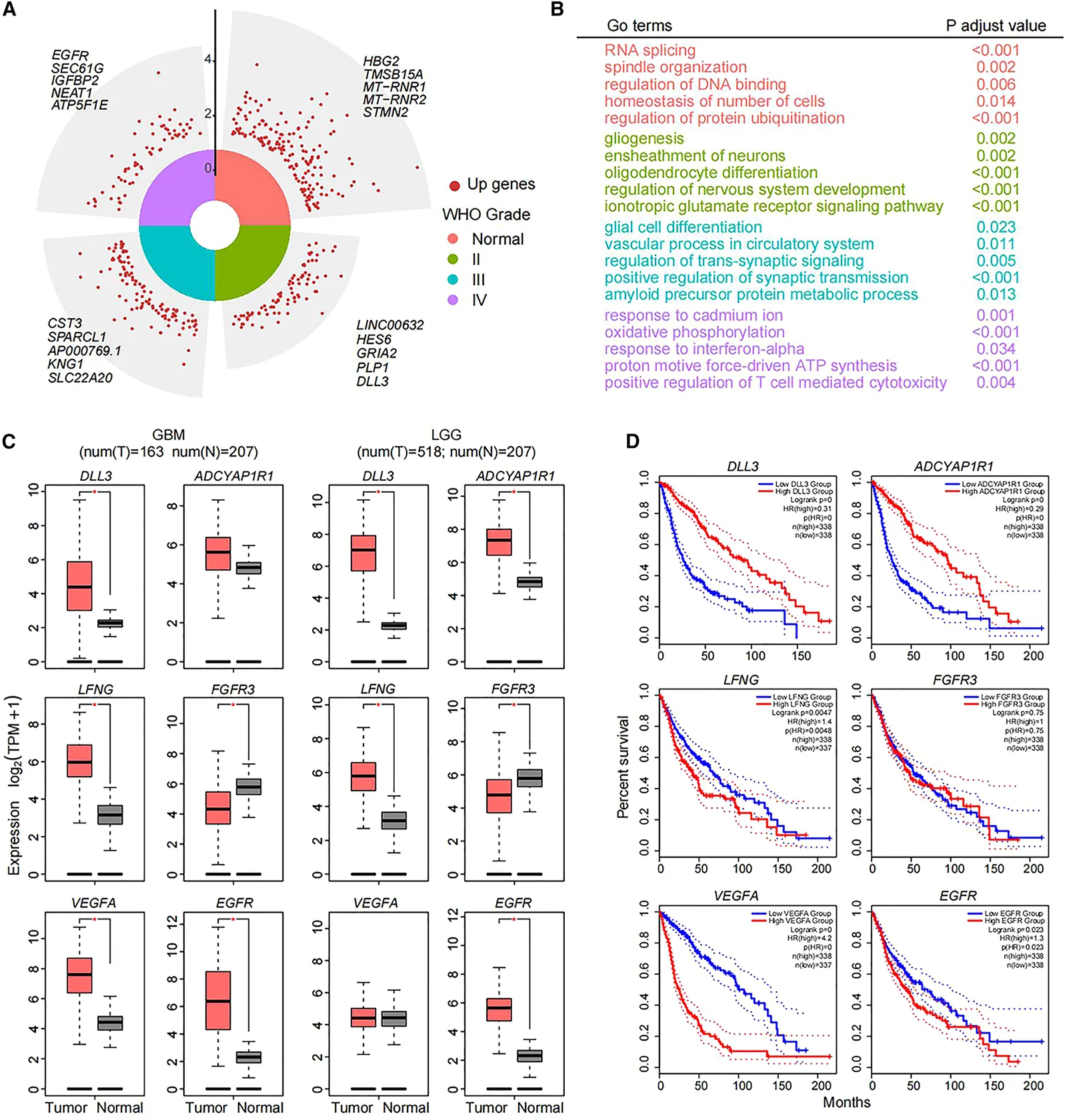
Identification of communication-driven genes associated with glioma malignancy and prognosis (A) Circular plot showing upregulated genes across different WHO grades in GSCs/NSCs. (B) GO enrichment analysis of upregulated genes of different WHO grades in GSCs/NSCs. (C) Boxplots showing expression levels of six candidate genes (*DLL3*, ADCYAP1R1, *LFNG*, *FGFR3*, *VEGFA*, and *EGFR*) in tumor versus normal tissues in GBM and LGG cohorts. One-way ANOVA was used in (C). ∗*p* < 0.05. (D) Kaplan-Meier survival curves stratified by high and low expression levels of the six candidate genes.

### DLL3 is functionally required for glioma cell viability and proliferation

Among the six candidate genes, *DLL3* emerged as a prominent communication-driven regulator and was therefore selected for functional validation. Four short hairpin RNA (shRNA) constructs targeting *DLL3* were designed and evaluated, and two constructs with high knockdown efficiency were selected based on western blot analysis (Figures 5A and 5B). LN229 glioma cells were infected with lentiviruses carrying *DLL3* shRNA, and stable knockdown cell lines were established. Bright-field microscopy revealed that *DLL3*-depleted cells exhibited a marked reduction in cell density compared with control cells, with the difference becoming more pronounced over time (Figure 5C). Consistently, CCK-8 assays demonstrated that *DLL3* knockdown significantly impaired cell viability and proliferative capacity (Figure 5D), indicating that *DLL3* is required for sustained glioma cell growth.

**Figure 5:**
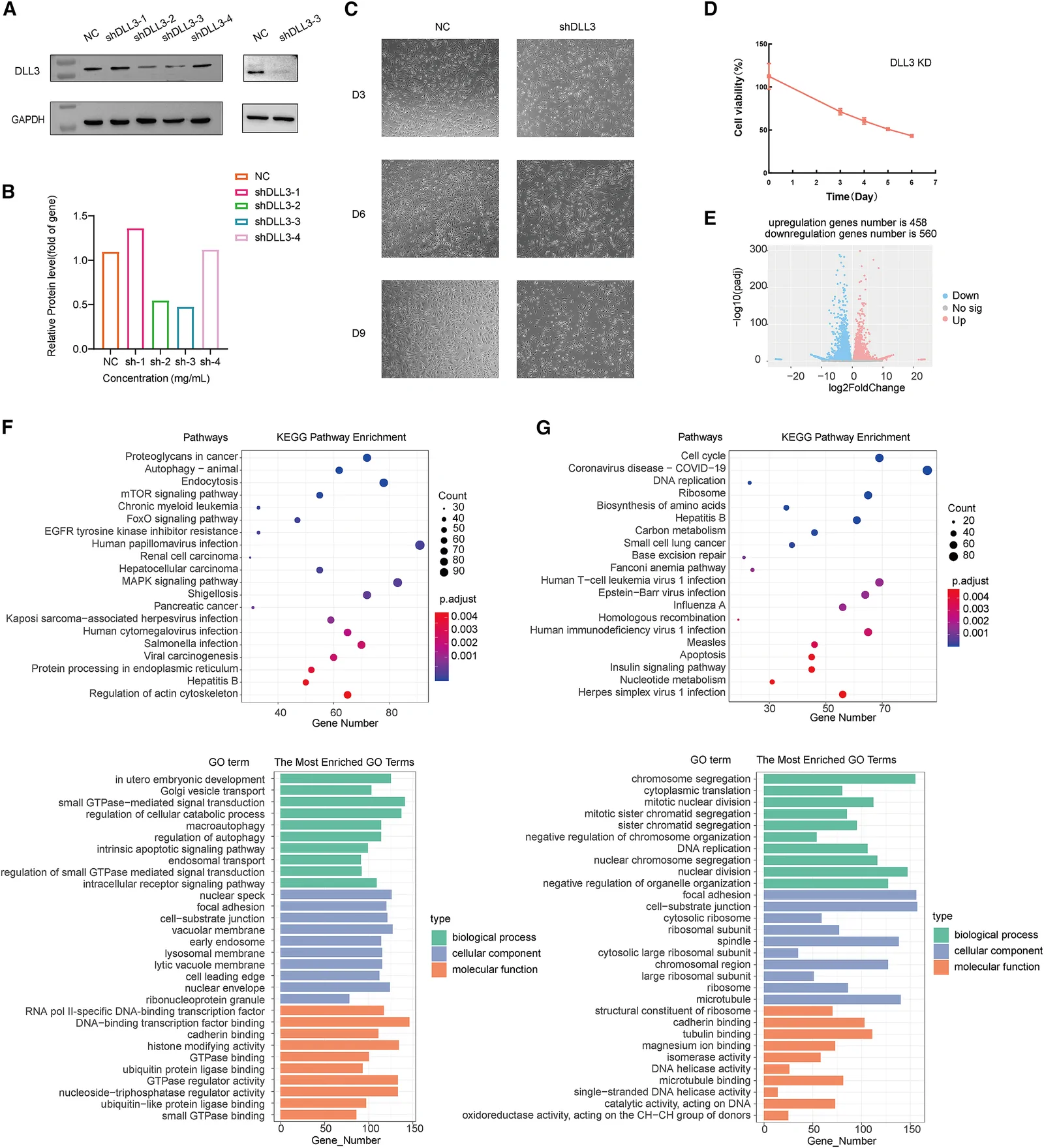
Experimental validation of DLL3 function in glioma cell proliferation and transcriptomic regulation (A and B) Western blot analysis confirming efficient knockdown of *DLL3* in glioma cells. (C) Representative phase-contrast images showing morphological changes in control (NC) and *DLL3*-knockdown (shDLL3) cells at days 3, 6, and 9. (D) Cell viability assay demonstrating reduced proliferation of glioma cells following *DLL3* knockdown over time. (E) Volcano plot showing differentially expressed genes upon *DLL3* knockdown. (F) KEGG pathway enrichment analysis of upregulated genes following *DLL3* silencing. (G) KEGG pathway enrichment analysis of downregulated genes following *DLL3* silencing.

### DLL3 depletion induces transcriptomic reprogramming and suppresses cell cycle-related pathways

To explore the molecular mechanisms underlying the growth-suppressive effects of *DLL3* knockdown, RNA-seq was performed on control and *DLL3*-depleted LN229 cells. Differential expression analysis identified 458 upregulated and 560 downregulated genes upon *DLL3* depletion (Figure 5E). Functional enrichment analysis of upregulated genes revealed significant enrichment in metabolic pathways and signal transduction processes (Figure 5F), whereas downregulated genes were predominantly enriched in pathways related to cell cycle regulation and proliferation (Figure 5G). Gene Ontology (GO) analysis further indicated that downregulated genes were associated with biological processes such as spindle organization, DNA replication, and cell division. These results suggest that *DLL3* supports glioma cell proliferation by maintaining cell cycle-related transcriptional programs, and that its depletion leads to widespread transcriptional reprogramming and suppression of proliferative signaling.

## Discussion

Glioma progression is increasingly understood as a multilevel process shaped by dynamic interactions between malignant cells and the surrounding TME.^35^^,^^36^ Rather than being solely driven by cell-autonomous genetic alterations, glioma evolution is orchestrated by reciprocal signaling between tumor cells, immune populations, glial cells, vascular components, and neural elements.^37^^,^^38^ In this study, we integrated large-scale single-cell transcriptomic profiling with intercellular communication inference to systematically delineate the cellular ecosystem and signaling architecture of glioma across disease stages. By identifying six communication-associated genes with prognostic relevance and functionally validating *DLL3* as a regulator of glioma proliferation, our work establishes a mechanistic link between microenvironmental crosstalk, transcriptional reprogramming, and tumor progression. This integrative framework provides a new perspective on glioma biology by positioning intercellular communication as a central driver of malignancy rather than a secondary consequence of tumor growth.

A defining feature of glioma revealed by our analysis is the profound cellular heterogeneity and stage-dependent remodeling of the TME. Through comprehensive single-cell profiling, we observed marked shifts in the proportions of malignant cells, myeloid populations, astrocytes, oligodendrocyte lineage cells, endothelial cells, and neurons during glioma progression. Importantly, these changes were not limited to expansion of tumor cells but involved coordinated reorganization of multiple non-malignant compartments. The depletion of neuronal populations, remodeling of oligodendrocyte progenitors, activation of reactive astrocytes, and expansion of vascular elements collectively indicate a transition from a homeostatic neural environment to a tumor-supportive niche. Such ecosystem-level remodeling is consistent with the emerging concept of cancer as a disease of tissue organization, in which disruption of normal cellular hierarchies and interactions creates permissive conditions for malignant growth. In the context of the central nervous system, where cellular identity and connectivity are tightly regulated, this distortion of the neural microenvironment may have particularly profound consequences for disease progression.^39^

Beyond cellular composition, our study highlights extensive rewiring of intercellular communication networks during glioma evolution. By inferring ligand-receptor-transcription factor interactions, we demonstrated that both the complexity and intensity of cell-cell signaling increase with tumor grade, particularly among glioma cells, myeloid cells, astrocytes, and vascular populations. This progressive intensification of cross-talk suggests that glioma progression is driven by network-level signaling reprogramming rather than isolated cellular events. Enhanced communication may establish feedforward loops that reinforce malignant phenotypes, promote immune suppression, and sustain metabolic adaptation. For example, reciprocal signaling between tumor cells and immune cells can facilitate immunosuppressive polarization, while interactions with astrocytes and endothelial cells may support invasion and angiogenesis.^40^^,^^41^^,^^42^ These findings align with and extend previous reports of neuron-glioma and immune-glioma cross-talk, underscoring that glioma is embedded within a highly interconnected cellular network. Importantly, this network perspective shifts the focus from individual pathways to the emergent properties of signaling systems, emphasizing that malignant behavior arises from coordinated interactions across cell types.

By integrating differential expression analysis with communication network inference, we identified six genes *DLL3*, *ADCYAP1R1*, *LFNG*, *FGFR3*, *VEGFA*, and *EGFR* that are embedded within intercellular signaling networks and associated with clinical outcomes. This integrative approach enabled us to prioritize genes that are not only dysregulated but also functionally positioned at communication interfaces. Among these candidates, *DLL3* emerged as a particularly intriguing regulator. *DLL3* is a non-canonical ligand of the Notch pathway, traditionally implicated in embryonic development and neurogenesis, and is aberrantly expressed in neuroendocrine tumors such as small cell lung cancer.^43^^,^^44^^,^^45^ However, its role in glioma has remained poorly characterized. Our data reveal that *DLL3* is required for glioma cell viability and proliferation and that its depletion leads to widespread transcriptional reprogramming, particularly suppression of cell cycle-related pathways. These findings suggest that *DLL3* may function as a context-dependent signaling modulator that integrates microenvironmental cues and sustains proliferative programs in malignant cells. Notably, pathway enrichment results revealed that the transcriptional reprogramming triggered by *DLL3* knockdown was predominantly concentrated in cell cycle-related pathways, with no significant enrichment of canonical Notch downstream target gene sets observed in our analysis. These findings suggest that *DLL3* regulates glioma proliferation mainly through cell cycle modulation rather than overt activation of classical Notch signaling cascades.

Nevertheless, the involvement of *DLL3* in glioma biology remains conceptually intriguing given its established association with the Notch signaling family and the critical role of developmental signaling pathways in NSC maintenance and tumor progression. In the developing nervous system, Notch pathway components regulate cell fate decisions, proliferation, and differentiation, ensuring proper tissue patterning and homeostasis. Reactivation or dysregulation of these developmental programs is a recurrent theme in cancer.^46^^,^^47^^,^^48^ Our findings raise the possibility that *DLL3*-mediated signaling may contribute to the maintenance of stem-like states and proliferative capacity in glioma cells, potentially linking developmental pathways to tumor progression. Moreover, the embedding of *DLL3* within communication networks suggests that its function may be shaped by interactions with surrounding cells, rather than operating solely as an intrinsic oncogenic driver. This context-dependent behavior is consistent with emerging views that signaling molecules can exert distinct effects depending on cellular context and network topology.

Based on our data, we propose a conceptual model in which glioma progression is driven by coordinated remodeling of the cellular ecosystem and intercellular communication networks. As malignant cells expand and the microenvironment becomes increasingly distorted, signaling interactions among glioma cells, immune populations, astrocytes, and vascular cells intensify and diversify. Within this rewired network, *DLL3* may act as a key regulatory node that integrates extracellular signals and maintains transcriptional programs required for cell cycle progression and proliferation. Such a model emphasizes that glioma malignancy is not merely the result of autonomous tumor cell behavior but emerges from dynamic and reciprocal interactions between malignant and non-malignant cells. By framing glioma progression as a network-driven process, this model provides a mechanistic basis for understanding how microenvironmental signals are translated into intracellular programs that drive tumor growth and resistance.

From a translational perspective, our findings have several important implications. First, the identification of *DLL3* as a functional regulator of glioma proliferation suggests that it may represent a potential therapeutic target, either directly or through modulation of its associated signaling pathways. Therapeutic strategies targeting *DLL3* have already been explored in other malignancies,^49^^,^^50^ raising the possibility that similar approaches could be adapted for glioma. Second, the communication networks uncovered in this study highlight intercellular cross-talk as a potential vulnerability of the tumor ecosystem. Disrupting key signaling interactions may destabilize tumor-supportive niches and enhance the efficacy of existing therapies. Third, our integrative framework combining single-cell profiling and communication inference provides a generalizable strategy to identify communication-driven regulators in other tumor types, offering a blueprint for dissecting complex microenvironmental interactions.

Several limitations of this study should be acknowledged. First, our single-cell analyses were based on publicly available datasets, which may introduce variability related to sample processing and sequencing platforms. Second, functional validation was performed in a single glioma cell line, and additional *in vitro* and *in vivo* models will be required to fully elucidate the role of *DLL3* in glioma biology. Therefore, we selected the LN229 cell line for functional validation primarily due to its favorable lentiviral transfection efficiency, which facilitates the effective knockdown of *DLL3* and reliable detection of subsequent functional changes. Third, intercellular communication networks were inferred computationally and therefore represent predicted interactions rather than direct experimental measurements. Integration of spatial transcriptomics, multiplex imaging, and functional perturbation studies will be essential to validate and refine these networks. Despite these limitations, our study provides a systematic and mechanistic perspective on glioma progression and establishes intercellular communication and *DLL3* as critical components of the malignant microenvironment.

In conclusion, by integrating single-cell transcriptomics, intercellular communication analysis, and functional validation, our work reveals glioma as a disease driven by ecosystem-level remodeling and network-level signaling reprogramming. The identification of *DLL3* as a communication-associated regulator of glioma proliferation highlights the importance of context-dependent signaling in tumor biology and opens new avenues for therapeutic intervention. More broadly, our findings underscore the value of viewing cancer through the lens of cellular ecosystems and communication networks, offering a conceptual framework that may be applicable to a wide range of malignancies.

## Materials and methods

### Data collection and processing of bulk and scRNA-seq data

All datasets of the section were obtained from public databases only. Bulk RNA-seq data of glioma, including GBM and LGG, were obtained from The Cancer Genome Atlas (TCGA) and Genotype-Tissue Expression (GTEx) databases. scRNA-seq datasets were retrieved from the Gene Expression Omnibus (GEO) under the accession numbers GSE103224, GSE117891, GSE134355, and GSE182109, comprising a total of 36 samples from tumor, peritumoral, normal adult brain, and normal fetal brain tissues. Raw unique molecular identifier (UMI) count matrices were processed using Seurat (version 4.0.5).^51^ Cells expressing fewer than 100 genes or more than 7,000 genes, as well as cells with mitochondrial gene expression exceeding 20%, were excluded. Genes expressed in fewer than 10 cells were filtered out. After quality control, a total of 246,594 high-quality cells were retained for downstream analyses. Batch effects among different datasets were corrected using the ACC algorithm. The top 2,000 highly variable genes were selected for principal component analysis (PCA), and the first 30 principal components were used for uniform manifold approximation and projection (UMAP) dimensionality reduction. Cell clustering was performed using the FindClusters function with a resolution of 0.5, resulting in 28 initial clusters. Cell types were annotated based on canonical marker genes reported in the literature, yielding eight major cell populations. Cells with low or absent *PTPRC* (*CD45*) expression were extracted for secondary reclustering to enrich for neural-lineage and stromal populations, resulting in 33 clusters, which were subsequently annotated into 12 cell types.

### Inference of CNVs in single cells

To distinguish malignant and non-malignant cells, CNV analysis was performed on low or absent *PTPRC* (*CD45*) expression cells using the CopyKAT package (version 1.1.0). Cells were classified as malignant or non-malignant based on inferred CNV profiles, and the distribution of malignant and non-malignant cells across different glioma stages was assessed.

### Differential expression analysis and functional enrichment

DEGs for GSCs/NSCs were identified using the FindAllMarkers function implemented in Seurat with the DESeq2 method. All stage-specific DEGs (156 at normal stage, 83 at stage II, 105 at stage III, and 63 at stage IV) were identified using the criteria of adjusted *p*-value <0.05 and |log2 fold change| > 1. The full set of these DEGs from each stage was then intersected with a curated list of cell-cell communication-related genes. After intersection, genes that appeared in at least one stage were retained, and a final set of six common genes was obtained after removing redundancies and applying a consistency filter. Functional enrichment analyses, including GO and Kyoto Encyclopedia of Genes and Genomes (KEGG) pathway analyses, were performed using Metascape to investigate the biological functions and pathways associated with the stage-specific DEGs.

### Cell-cell communication analysis

Cell-cell communication analysis was conducted using CellCall. Cell type annotations derived from the Seurat workflow were used as input. The TransCommuProfile function was applied to infer ligand-receptor interaction probabilities in different glioma stages. Cell-cell communication probabilities were displayed using the ViewInterCircos function to identify ligand-receptor signaling associated with intercellular communication in the glioma microenvironment.

### Survival analysis

Candidate genes were identified by intersecting stage-specific DEGs with a curated list of cell-cell communication-related genes, followed by redundancy removal and consistency filtering. The expression levels of these six genes in GBM and LGG were analyzed using GEPIA 2.0 based on TCGA and GTEx datasets. Kaplan-Meier survival analysis was performed to evaluate the prognostic significance of each gene in the TCGA GBM and LGG. Use the median as a criterion to distinguish between the high- and low-expression conditions of the groups.

### shRNA design and plasmid construction

Four distinct shRNA sequences targeting the human *DLL3* mRNA (NCBI Gene ID: 10683) were designed. The corresponding DNA oligonucleotides were then synthesized. The annealed oligonucleotide duplexes were ligated into the pLKO.1 vector, a lentiviral backbone vector carrying a hygromycin B resistance cassette for stable cell selection, which had been linearized by digestion with *Age*I and *Eco*RI restriction enzymes, to generate the pLKO.1-shDLL3 knockdown constructs. The correct insertion and sequence of each shRNA cassette in all final constructs were confirmed by Sanger sequencing.

### Lentiviral packaging and generation of stable knockdown cell lines

For lentiviral production, HEK293T cells were seeded and cultured for 24 h to achieve approximately 60%–70% confluence prior to transfection. The cells were then transfected with a plasmid mixture consisting of 10 μg pLKO.1-shDLL3, 7.5 μg psPAX2, and 2.5 μg pMD2.G, using polyethylenimine (PEI) as the transfection reagent (80 μL PEI in 320 μL Opti-MEM). Twelve hours post-transfection, the medium was replaced with fresh complete growth medium. The viral supernatant was harvested at both 24 and 48 h after transfection, pooled, and used for subsequent infection.

For infection, LN229 glioma cells were similarly seeded to reach 60%–70% confluence 24 h in advance. The cells were washed twice with Dulbecco’s phosphate-buffered saline (DPBS) and then incubated with the harvested viral supernatant supplemented with polybrene at a final dilution of 1:1,000. To select for stably transduced cells, fresh medium containing hygromycin B (at a 1:500 dilution) was added 24 h post-infection. Selection was maintained for 3 days with a medium change every 1.5 days, after which stable polyclonal *DLL3*-knockdown cell populations were established.

### Cell viability assay

Cell viability was assessed using the Cell Counting Kit-8 (CCK-8) assay. Control (Ctrl) and DLL3 knockdown (KD) LN229 cells were seeded at a density of 5,000 cells per well in 96-well plates (in 100 μL of culture medium per well), with six replicates per group. At 0, 3, 4, 5, and 6 days, 10 μL of CCK-8 reagent was added to each well and incubated at 37 °C for 2 h in the dark. Absorbance was measured at 450 nm using a microplate reader. Cell viability was calculated based on optical density (OD) values. Statistical analysis was performed using two-way analysis of variance (ANOVA) in GraphPad Prism 9.0.

### RNA-seq and transcriptomic analysis

Total RNA was extracted from control and DLL3 knockdown LN229 cells (two biological replicates) using TRIzol reagent. mRNA was then enriched using VAHTS mRNA Capture Beads (Ultrapure) (RN702) for library construction with the VAHTS Universal V8 RNA-seq Library Prep Kit for Illumina (NR605). Briefly, the mRNA was fragmented and reverse-transcribed to generate first-strand cDNA. Second-strand synthesis, end repair, and dA-tailing were performed in a single step, followed by adapter ligation and PCR amplification. The resulting libraries were size-selected using magnetic beads and sequenced to a depth of 10 Gb per sample. Differential expression analysis was performed using DESeq2, and the results were visualized with volcano plots. Functional enrichment analysis of the DEGs was conducted using the KEGG and GO databases.

### Western blot analysis

Equal amounts of protein were separated on 10% SDS-polyacrylamide gels and transferred onto nitrocellulose membranes using a wet transfer system at 100 V for 90 min. Membranes were blocked with 5% non-fat milk in TBST and incubated overnight at 4°C with primary antibodies against *DLL3* (1:200; A18108; Abclonal) and *GAPDH* (1:10,000; 60004-1-Ig; Proteintech) as a loading control. After washing with TBST, membranes were incubated with the corresponding HRP-conjugated secondary antibodies (1:5,000) at room temperature for 2 h. Protein bands were detected using an enhanced chemiluminescence (ECL) system and visualized with a multispectral imaging system.

## Data and code availability

The bulk sequencing dataset generated in this study is publicly available from the National Genomics Data Center: HRA016394. The scRNA-seq datasets analyzed in this study are publicly available from the Gene Expression Omnibus (GEO) database under the accession numbers: GSE103224, GSE117891, GSE134355, and GSE182109. Bulk RNA-seq data were obtained from The Cancer Genome Atlas (TCGA) and the Genotype-Tissue Expression (GTEx) databases. RNA-seq data generated in this study are available from the corresponding author upon reasonable request.

## Acknowledgments

We thank Professor Lin Ning for his constructive discussions on the research content and manuscript preparation. This work was supported by the 10.13039/501100001809National Natural Science Foundation of China (62202069), the 10.13039/501100018542Natural Science Foundation of Sichuan Province (2026NSFSC0422). This study did not involve any human participants or animal experiments. All analyses were conducted using publicly available datasets and established glioma cell lines.

## Author contributions

H.Q., J.H., and Y.Z. conceived and designed the study. L.X., X.P., and H.W. performed the bioinformatics analyses. L.X. and H.W. conducted the cell experiments. Z.Y. and J.H. assisted with data analysis and interpretation. L.X., J.H., Y.Z., and H.Q. drafted the manuscript. All authors reviewed, revised, and approved the final manuscript.

## Declaration of interests

The authors declare no competing interests.

## Declaration of generative AI and AI-assisted technologies in the writing process

During the preparation of this manuscript, the authors utilized DeepSeek R1 to enhance the language and readability. Following the use of this service, the authors thoroughly reviewed and revised the content as necessary, and take full responsibility for the final version of the publication.

## Supplemental information

Table S1. Ligand-receptor interaction networks in normal, WHO grade II, WHO grade III, and WHO grade IV glioma samples

Table S2. DEGs and GO enrichment analysis in glioma samples of different grades

## References

[1] Omuro A., DeAngelis L.M. (2013). Glioblastoma and other malignant gliomas: a clinical review. JAMA.

[2] Wang L.M., Englander Z.K., Miller M.L., Bruce J.N. (2023). Malignant Glioma. Adv. Exp. Med. Biol..

[3] Weller M., Wen P.Y., Chang S.M., Dirven L., Lim M., Monje M., Reifenberger G. (2024). Glioma. Nat. Rev. Dis. Primers.

[4] Schaff L.R., Mellinghoff I.K. (2023). Glioblastoma and Other Primary Brain Malignancies in Adults: A Review. JAMA.

[5] Lan Z., Li X., Zhang X. (2024). Glioblastoma: An Update in Pathology, Molecular Mechanisms and Biomarkers. Int. J. Mol. Sci..

[6] Zhang X., Ma H., Gao Y., Liang Y., Du Y., Hao S., Ni T. (2024). The Tumor Microenvironment: Signal Transduction. Biomolecules.

[7] Shah D.D., Chorawala M.R., Raghani N.R., Patel R., Fareed M., Kashid V.A., Prajapati B.G. (2025). Tumor microenvironment: recent advances in understanding and its role in modulating cancer therapies. Med. Oncol..

[8] Lyu Y., Yang H., Chen L. (2022). Metabolic regulation on the immune environment of glioma through gut microbiota. Semin. Cancer Biol..

[9] Barthel L., Hadamitzky M., Dammann P., Schedlowski M., Sure U., Thakur B.K., Hetze S. (2022). Glioma: molecular signature and crossroads with tumor microenvironment. Cancer Metastasis Rev..

[10] Mishchenko T.A., Turubanova V.D., Gorshkova E.N., Krysko O., Vedunova M.V., Krysko D.V. (2024). Glioma: bridging the tumor microenvironment, patient immune profiles and novel personalized immunotherapy. Front. Immunol..

[11] Jayaram M.A., Phillips J.J. (2024). Role of the Microenvironment in Glioma Pathogenesis. Annu. Rev. Pathol..

[12] Cvitković J., Tan W.L., Jiang T., Zhao Z. (2025). Glioma tumor microenvironment and immunotherapy: past, present, and future. Biomark. Res..

[13] Liu T., Qiao H., Ren L., Ye X., Zou Q., Zhang Y. (2025). Deciphering cancer therapy resistance via patient-level single-cell transcriptomics with CellResDB. Commun. Biol..

[14] Venkatesh H.S., Johung T.B., Caretti V., Noll A., Tang Y., Nagaraja S., Gibson E.M., Mount C.W., Polepalli J., Mitra S.S. (2015). Neuronal Activity Promotes Glioma Growth through Neuroligin-3 Secretion. Cell.

[15] Taylor K.R., Barron T., Hui A., Spitzer A., Yalçin B., Ivec A.E., Geraghty A.C., Hartmann G.G., Arzt M., Gillespie S.M. (2023). Glioma synapses recruit mechanisms of adaptive plasticity. Nature.

[16] Tang Q., Yu Y., Shen M., Zhang L., Jia X., Kang J. (2025). cncFinder: a graph-attention-network-based interpretable learning model to identify bifunctional long non-coding RNAs. Mol. Ther. Nucleic Acids.

[17] Ausejo-Mauleon I., Labiano S., de la Nava D., Laspidea V., Zalacain M., Marrodán L., García-Moure M., González-Huarriz M., Hervás-Corpión I., Dhandapani L. (2023). TIM-3 blockade in diffuse intrinsic pontine glioma models promotes tumor regression and antitumor immune memory. Cancer Cell.

[18] Guo X., Sui R., Piao H. (2023). Exosomes-mediated crosstalk between glioma and immune cells in the tumor microenvironment. CNS Neurosci. Ther..

[19] Karimi E., Yu M.W., Maritan S.M., Perus L.J.M., Rezanejad M., Sorin M., Dankner M., Fallah P., Doré S., Zuo D. (2023). Single-cell spatial immune landscapes of primary and metastatic brain tumours. Nature.

[20] Wan S., Moure U.A.E., Liu R., Liu C., Wang K., Deng L., Liang P., Cui H. (2022). Combined bulk RNA-seq and single-cell RNA-seq identifies a necroptosis-related prognostic signature associated with inhibitory immune microenvironment in glioma. Front. Immunol..

[21] Chen R., Wu W., Liu T., Zhao Y., Wang Y., Zhang H., Wang Z., Dai Z., Zhou X., Luo P. (2022). Large-scale bulk RNA-seq analysis defines immune evasion mechanism related to mast cell in gliomas. Front. Immunol..

[22] Fan K., Wei Y., Ou Y., Gong J. (2023). Integrated analysis of multiple methods reveals characteristics of the immune microenvironment in medulloblastoma. Brain Tumor Pathol..

[23] Ahmed Z., Shahzadi K., Jin Y., Li R., Momanyi B.M., Zulfiqar H., Ning L., Lin H. (2024). Identification of RNA-dependent liquid-liquid phase separation proteins using an artificial intelligence strategy. Proteomics.

[24] Tirosh I., Suvà M.L. (2018). Dissecting human gliomas by single-cell RNA sequencing. Neuro Oncol..

[25] Hernández Martínez A., Madurga R., García-Romero N., Ayuso-Sacido Á. (2022). Unravelling glioblastoma heterogeneity by means of single-cell RNA sequencing. Cancer Lett..

[26] Fu Y., Yi Y., Shao Y., Jiang J., Deng Q. (2025). Single-cell and spatial transcriptomic insights into glioma cellular heterogeneity and metabolic adaptations. Front. Immunol..

[27] Cheng C., Chen W., Jin H., Chen X. (2023). A Review of Single-Cell RNA-Seq Annotation, Integration, and Cell-Cell Communication. Cells.

[28] Kürten C.H.L., Kulkarni A., Cillo A.R., Santos P.M., Roble A.K., Onkar S., Reeder C., Lang S., Chen X., Duvvuri U. (2021). Investigating immune and non-immune cell interactions in head and neck tumors by single-cell RNA sequencing. Nat. Commun..

[29] Zhang Y., Yang Y., Ren L., Zhan M., Sun T., Zou Q., Zhang Y. (2024). Predicting intercellular communication based on metabolite-related ligand-receptor interactions with MRCLinkdb. BMC Biol..

[30] Cui X., Liu S., Song H., Xu J., Sun Y. (2025). Single-cell and spatial transcriptomic analyses revealing tumor microenvironment remodeling after neoadjuvant chemoimmunotherapy in non-small cell lung cancer. Mol. Cancer.

[31] Ochocka N., Segit P., Walentynowicz K.A., Wojnicki K., Cyranowski S., Swatler J., Mieczkowski J., Kaminska B. (2021). Single-cell RNA sequencing reveals functional heterogeneity of glioma-associated brain macrophages. Nat. Commun..

[32] Xie Y., Yang F., He L., Huang H., Chao M., Cao H., Hu Y., Fan Z., Zhai Y., Zhao W. (2024). Single-cell dissection of the human blood-brain barrier and glioma blood-tumor barrier. Neuron.

[33] Xu H., Chai H., Chen M., Zhu R., Jiang S., Liu X., Wang Y., Chen J., Wei J., Mao Y., Shi Z. (2024). Single-cell RNA sequencing identifies a subtype of FN1 + tumor-associated macrophages associated with glioma recurrence and as a biomarker for immunotherapy. Biomark. Res..

[34] Zhang Y., Liu T., Hu X., Wang M., Wang J., Zou B., Tan P., Cui T., Dou Y., Ning L. (2021). CellCall: integrating paired ligand-receptor and transcription factor activities for cell-cell communication. Nucleic Acids Res..

[35] Jang H.J., Park J.W. (2025). Microenvironmental Drivers of Glioma Progression. Int. J. Mol. Sci..

[36] Song K.W., Lim M., Monje M. (2025). Complex neural-immune interactions shape glioma immunotherapy. Immunity.

[37] He J., Yan X., Hu S. (2025). Glioma stem cells: drivers of tumor progression and recurrence. Stem Cell Res. Ther..

[38] Ramachandran R., Jeans A.F. (2025). Breaking Down Glioma-Microenvironment Crosstalk. Neuroscientist.

[39] Wu T., Zhao P., Chen Z., Ying T., Nie Z., Hua W., Li H., Mao Y., Bu W. (2025). Neuro-Cancer Interactions Shape Glioma Intratumoral Heterogeneity. Adv. Sci..

[40] Liu J., Piranlioglu R., Ye F., Shu K., Lei T., Nakashima H. (2023). Immunosuppressive cells in oncolytic virotherapy for glioma: challenges and solutions. Front. Cell. Infect. Microbiol..

[41] De Domenico P., Gagliardi F., Roncelli F., Snider S., Mortini P. (2025). Tumor-infiltrating and circulating B cells mediate local and systemic immunomodulatory mechanisms in Glioblastoma. J. Neuro Oncol..

[42] Musatova O.E., Rubtsov Y.P. (2023). Effects of glioblastoma-derived extracellular vesicles on the functions of immune cells. Front. Cell Dev. Biol..

[43] Nilsson I., Su E.J., Fredriksson L., Sahlgren B.H., Bagoly Z., Moessinger C., Stefanitsch C., Ning F.C., Zeitelhofer M., Muhl L. (2024). Thrombolysis exacerbates cerebrovascular injury after ischemic stroke via a VEGF-B dependent effect on adipose lipolysis. bioRxiv.

[44] Peddio A., Pietroluongo E., Lamia M.R., Luciano A., Caltavituro A., Buonaiuto R., Pecoraro G., De Placido P., Palmieri G., Bianco R. (2024). DLL3 as a potential diagnostic and therapeutic target in neuroendocrine neoplasms: A narrative review. Crit. Rev. Oncol. Hematol..

[45] Wang H., Zheng T., Xu D., Sun C., Huang D., Liu X. (2025). Targeting DLL3: Innovative Strategies for Tumor Treatment. Pharmaceutics.

[46] Kipper F.C., Kieran M.W., Thomas A., Panigrahy D. (2022). Notch signaling in malignant gliomas: supporting tumor growth and the vascular environment. Cancer Metastasis Rev..

[47] Kumari L., Mishra L., Sharma Y., Chahar K., Kumar M., Patel P., Gupta G.D., Kurmi B.D. (2024). NOTCH Signaling Pathway: Occurrence, Mechanism, and NOTCH-Directed Therapy for the Management of Cancer. Cancer Biother. Radiopharm..

[48] Xia R., Xu M., Yang J., Ma X. (2022). The role of Hedgehog and Notch signaling pathway in cancer. Mol. Biomed..

[49] Rudin C.M., Reck M., Johnson M.L., Blackhall F., Hann C.L., Yang J.C.H., Bailis J.M., Bebb G., Goldrick A., Umejiego J., Paz-Ares L. (2023). Emerging therapies targeting the delta-like ligand 3 (DLL3) in small cell lung cancer. J. Hematol. Oncol..

[50] Ding J., Yeong C. (2024). Advances in DLL3-targeted therapies for small cell lung cancer: challenges, opportunities, and future directions. Front. Oncol..

[51] Wu F., Zhang Z., Wang M., Ma Y., Verma V., Xiao C., Zhong T., Chen X., Wu M., Yu J., Chen D. (2023). Cellular Atlas of Senescent Lineages in Radiation- or Immunotherapy-Induced Lung Injury by Single-Cell RNA-Sequencing Analysis. Int. J. Radiat. Oncol., Biol., Phys..

